# Effect of air-borne particle abrasion and tribochemical silica coating on surface characteristics, mechanical behavior, and bonding performance of 3D-printed resin-based materials

**DOI:** 10.1007/s00784-026-07123-z

**Published:** 2026-09-12

**Authors:** Rafael Dascanio, Bruna S.H. Tonin, Isabela Morelli Gama, Sofia Pantarotto, Milena Santos Medeiros, Mutlu Özcan

**Affiliations:** 1 Department of Restorative Dentistry, Piracicaba Dental School, University of Campinas, Piracicaba, São Paulo Brazil; 2 Clinic of Masticatory Disorders and Dental Biomaterials, Center for Dental Medicine, University of Zurich, Plattenstrasse 11, Zurich, 8032 Switzerland; 3 Department of Restorative Dentistry, Ribeirão Preto School of Dentistry, University of São Paulo, Ribeirão Preto, SP Brazil; 4 School of Dentistry, University of São Paulo, São Paulo, Brazil

**Keywords:** 3D-printing, Adhesion, Air-abrasion, Alumina, Dental materials, Prosthodontics, Silica coating, Surface conditioning

## Abstract

**Objectives:**

This study evaluated how air-abrasion material, application time, and aging affect the surface characteristics, mechanical behavior, and bonding performance of three-dimensionally (3D) printed resin.

**Methods:**

Specimens were treated with aluminum oxide (Al₂O₃) or tribochemical silica coating (SiO₂) for 5, 10, and 15 s (50 μm, 2 bar). Surface roughness (Ra), flexural strength, elastic modulus, and shear bond strength (SBS) were measured. Aging included 5,000 thermocycles. Data were analyzed using ANOVA and Tukey’s test (α = 0.05).

**Results:**

Roughness was significantly affected by the abrasive material, application time and their interaction (*p* < 0.001). Al₂O₃ produced a time-dependent increase in roughness, whereas SiO₂ remained stable across intervals. Flexural strength was significantly reduced in all treated groups relative to the untreated control (*p* < 0.001). Elastic modulus was affected by surface conditioning, while aging (*p* = 0.979) and interaction effects (*p* = 0.966) were not significant. SBS was significantly influenced by abrasive material and application time, but not by aging or interaction effects (*p* > 0.05). SiO₂ showed more stable bonding across time, while Al₂O₃ was time-dependent.

**Conclusion:**

Both surface-conditioning protocols improved bond strength. Tribochemical silica coating provided higher bond strength while more consistently preserving the mechanical properties of the printed resin, and a 10 s application was sufficient, as extending the treatment to 15 s provided no additional benefit under the conditions evaluated. Increased surface roughness did not necessarily result in improved bond strength.

**Clinical relevance:**

Tribochemical silica coating offers more predictable bonding outcomes for 3D-printed restorations, particularly when precise chairside application times cannot be guaranteed, thereby minimizing the risk of adhesive failure.

**Supplementary Information:**

The online version contains supplementary material available at https://doi.org/10.1007/s00784-026-07123-z.

## Introduction

Air-borne particle abrasion (hereon: air-abrasion) has been widely demonstrated to enhance bond strength to polymer-based dental materials, as consistently reported in both experimental and systematic studies [1–4]. This effect is primarily attributed to increased surface roughness and micromechanical interlocking, which improve interfacial retention and wettability [2, 5, 6]. However, despite this consolidated evidence, the procedure is often treated as a standardized step, with limited consideration of how variations in its parameters may influence material behavior beyond adhesion [7, 8].

Among these parameters, the duration of air-abrasion remains poorly explored in three-dimensionally (3D) printed resin-based materials. Different exposure times are expected to produce distinct surface and subsurface conditions, potentially affecting not only bonding effectiveness but also the mechanical performance of the substrate. As an inherently aggressive process, air-abrasion may induce defect accumulation, including microcracks and structural damage, particularly when exposure is prolonged [5, 9, 10]. While moderate surface modification may enhance adhesion, excessive treatment may shift the balance toward defect-driven weakening, compromising structural reliability. This issue is particularly relevant for additively manufactured resin-based materials. Due to their layer-by-layer fabrication, these materials exhibit intrinsic structural heterogeneity, including variations in degree of conversion, interlayer cohesion, and residual stresses [11–13]. Such features may increase their susceptibility to surface-induced damage, especially when additional modifications are introduced prior to bonding. In this context, surface conditioning should not be interpreted as a purely superficial modification, but rather as an extension of the material’s structural condition.

Among the available surface conditioning, aluminum oxide (Al₂O₃) air-abrasion and tribochemical silica coating (SiO₂) differ not only in their mechanisms but also in their implications for interfacial behavior [3, 14]. Al₂O₃ abrasion primarily promotes mechanical roughening through particle impact and material removal, whereas tribochemical silica coating combines mechanical interaction with chemical modification, enabling silane-mediated coupling and potentially improving chemical integration at the interface [2, 4, 8, 15, 16].

The duration of air-abrasion represents a critical variable within this balance. Increasing exposure time may enhance surface activation; however, it may also intensify damage accumulation. At shorter times, surface modification may be beneficial, improving wettability and micromechanical interlocking. With prolonged exposure, progressive defect formation may reach a threshold at which further roughening no longer translates into improved bonding but instead facilitates crack initiation and reduces resistance to loading [9, 10]. Although air-abrasion most studies have focused on isolated outcomes, such as bond strength or surface roughness, without considering their relationship with mechanical behavior and defect formation. Moreover, the combined effects of abrasive material, exposure time, and aging on multiple properties remain insufficiently explored, particularly in 3D-printed polymer materials [4].

Therefore, the aim of this study was to evaluate the influence of surface conditioning (Al₂O₃ vs. SiO₂), air-abrasion time (5, 10, and 15 s), and thermo-mechanical aging (before and after) on surface roughness, flexural strength, elastic modulus, and shear bond strength of 3D-printed resin-based materials. The null hypotheses tested were that (1) surface conditioning, (2) air-abrasion time, and (3) aging condition would not significantly affect the evaluated properties, and that no interaction would exist among these factors.

## Materials and methods

**Fig. 1:**
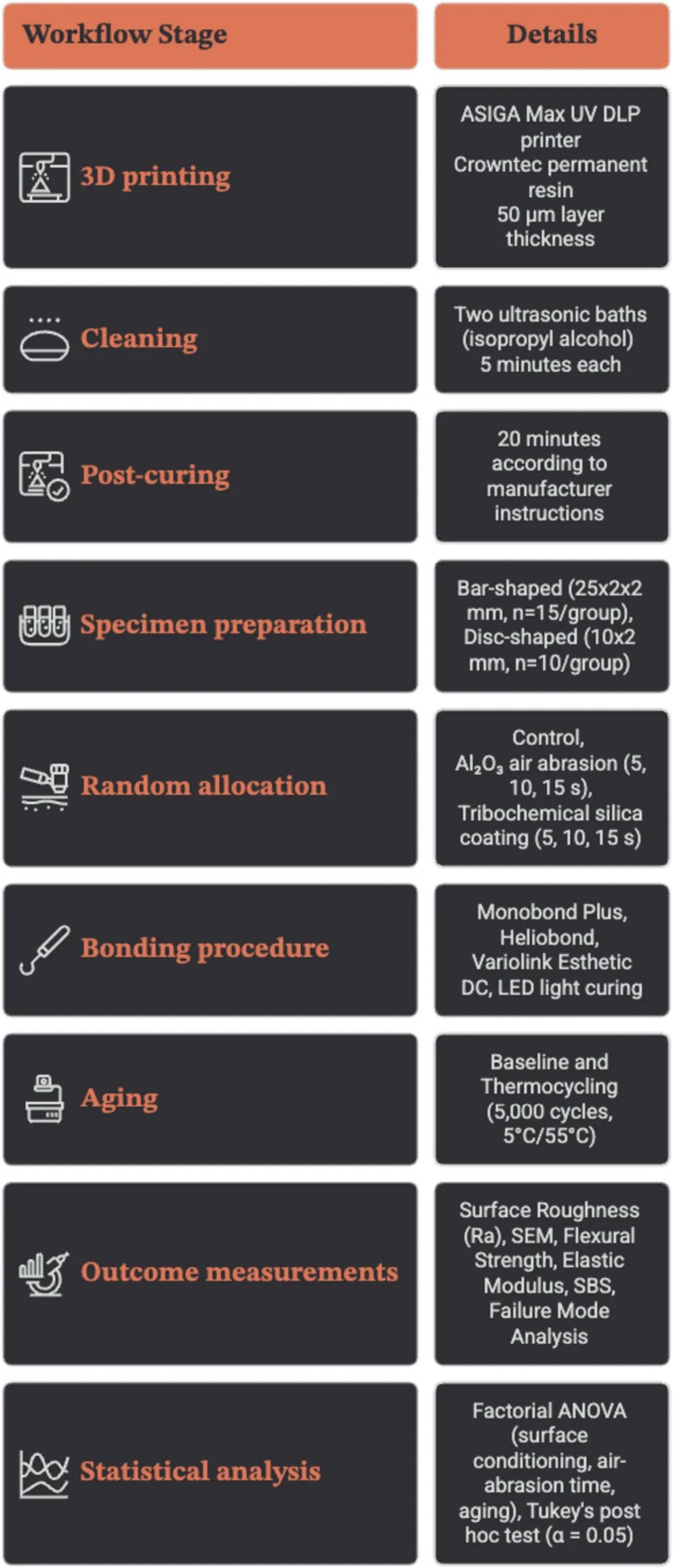
Study flow diagram

A fully crossed factorial design was adopted to evaluate the influence of surface conditioning, air-abrasion time, thermomechanical aging on the properties of 3D-printed resin-based materials. The independent variables were: (1) surface conditioning, including untreated control, aluminum oxide air-abrasion (Al₂O₃), and tribochemical silica coating (SiO₂); (2) air-abrasion time (5, 10, and 15 s); and (3) aging condition (before and after thermo-mechanical aging). The dependent variables were surface roughness (Ra), flexural strength, elastic modulus, and shear bond strength (SBS). For treated groups, factorial comparisons were performed considering abrasive material, air-abrasion time, and aging condition. The untreated control group was included as an additional reference for pairwise comparisons (Fig. 1).

### Sample size estimation

A sample size estimation was performed considering flexural strength as the primary outcome. The calculation was based on a fixed-effects factorial analysis of variance, with a significance level of 0.05, a statistical power of 80%, and an expected effect size of Cohen’s f = 0.40. The minimum estimated sample size was 93 specimens. To allow balanced allocation across the 14 experimental conditions, this number was rounded to 98 specimens, corresponding to at least seven specimens per condition. A total of 210 bar-shaped specimens were fabricated, with 15 specimens allocated to each experimental condition. Based on the final sample size, the achieved statistical power was approximately 98.1%.

### Specimen preparation, surface conditioning and cementation procedure

All specimens were additively manufactured using a digital light processing (DLP) 3D printer (ASIGA Max UV, ASIGA, Australia) with a methacrylate-based resin indicated for definitive restorations (Crowntec, Saremco Dental AG, Switzerland). Specimens were printed with a 0° build orientation (parallel to the build platform) and a layer thickness of 50 μm, following the manufacturer’s recommended printing parameters. After printing, specimens were cleaned in two consecutive ultrasonic baths containing isopropyl alcohol for 5 min each to remove uncured resin. Post-curing was then performed for 20 min using a post-curing unit (ELEGOO Mercury Plus, ELEGOO, Shenzhen, China), following the resin manufacturer’s recommended post-curing protocol.

Specimens were fabricated according to the requirements of each experimental test. For mechanical analysis, fifteen bar-shaped specimens per group (25 × 2 × 2 mm^3^) were prepared following ISO 4049:2019 for flexural strength and elastic modulus evaluation. Surface conditioning were performed at the central region of each bar. For surface and interfacial analyses, disc-shaped specimens (10 mm diameter × 2 mm thickness) were fabricated (*n* = 10 per group). These specimens were used for surface roughness, cementation (shear bond strength), and scanning electron microscopy (SEM) analysis (*n* = 2).

Specimens were randomly allocated to the following groups: untreated control, aluminum oxide air-abrasion (Al₂O₃), and tribochemical silica coating (SiO₂; SilJet, Danville Materials, USA). Air-abrasion with Al₂O₃ was performed using 50 μm particles at 2 bar pressure. During air abrasion, the distance between the nozzle and the specimen surface was standardized using a millimeter ruler as a reference instrument for keeping the distance of the nozzle to the surface standard. The air-abrasion handpiece was used in its standard configuration, which directs the abrasive particle stream at approximately 45° relative to the specimen surface, thereby ensuring a consistent application angle throughout the procedure. For the tribochemical silica coating group, silica-modified particles were applied under identical conditions. Application times were 5, 10, and 15 s, controlled using a digital timer. After treatment, all specimens were ultrasonically cleaned to remove residual particles. Following surface conditioning, a universal primer (Monobond Plus) was applied and allowed to react for 60 s. Subsequently, a thin layer of bonding resin (Heliobond) was applied without prior light curing to enhance surface wetting and provide a low-viscosity methacrylate-rich interfacial layer, facilitating copolymerization with the dual-cure resin cement. A dual-cure resin cement (Variolink Esthetic DC) was then used for bonding. The cement was applied using a transparent cylindrical mold with an internal diameter of 3 mm to standardize the bonding area. Excess cement was carefully removed, and light activation was performed for 30 s using a light-emitting diode (LED) curing unit (Bluephase, Ivoclar Vivadent). Specimens assigned to the aging condition were subjected to thermocycling using a thermocycling machine (Julabo GmbH, Seelbach, Germany). A total of 5,000 thermal cycles were performed between 5 °C and 55 °C, with a dwell time of 30 s in each bath and a transfer time of 5 s between baths, following the recommendations of ISO TR 11,405.

### Surface roughness (Ra)

Surface roughness (Ra) was measured using a 3D digital microscope (VK-X series, Keyence, Osaka, Japan). Measurements were performed under standardized conditions using a cutoff length (λc) of 1.25 mm and an evaluation length of 0.25 mm. For each specimen, six measurements were obtained (three in the horizontal and three in the vertical direction) to account for surface anisotropy. The mean value was used as the representative Ra for each specimen [10].

### Scanning electron microscopy (SEM)

The surface morphology of representative specimens from each experimental group was analyzed by SEM (TM4000Plus, Hitachi High-Tech Corporation, Tokyo, Japan). Specimens were gently air-dried and examined under low-vacuum conditions without conductive coating at an accelerating voltage of 15 kV. Micrographs were obtained at standardized magnifications (500×) to qualitatively assess surface topography and the presence of surface irregularities induced by the different treatments [10].

## Flexural strength and Elastic modulus

Flexural strength and elastic modulus were determined using a three-point bending test in a universal testing machine (ZwickRoell Z010, ZwickRoell GmbH, Ulm, Germany), following ISO 4049. Bar-shaped specimens (25 × 2 × 2 mm^3^) were positioned on a 20 mm support span and loaded at a crosshead speed of 0.5 mm/min until failure [10].

Flexural strength (σ, MPa) was calculated as:

$$\:{{\upsigma\:}}_{f}\text{}=\frac{3FL}{{2bh}^{2}}\text{}$$

where *F* is the maximum load at failure (N), *L* is the support span (mm), *b* is the specimen width (mm), and *h* is the specimen height (mm).

Elastic modulus (E, GPa) was obtained from the slope of the linear portion of the load–displacement curve obtained during the same test, according to ISO 4049, using the following equation:

$$\:\mathrm{E}=\frac{{FL}^{3}}{4{bh}^{3}d}$$

where *F* is the load within the elastic region (N), *L* is the support span (mm), *b* is the specimen width (mm), *h* is the specimen height (mm), and δ is the corresponding deflection (mm).

### Adhesion test

Shear bond strength (SBS) was evaluated using a universal testing machine (ZwickRoell Z010, ZwickRoell GmbH, Ulm, Germany) at a crosshead speed of 0.5 mm/min until failure. Prior to testing, each specimen was secured in a custom-made metal jig to ensure stable positioning and standardized alignment during load application. A chisel-shaped loading device was aligned parallel to the bonded interface and positioned in contact with the cement cylinder, as close as possible to the adhesive interface, in order to concentrate shear stresses at the bonded region. The test was performed under displacement-controlled conditions at a constant crosshead speed until failure. The maximum load at failure (Fmax, N) was recorded. Shear bond strength values were calculated by dividing the failure load by the bonded surface area and expressed in megapascals (MPa). A transparent cylindrical mold was used during cementation to standardize the bonding area, with an internal diameter (d) of 3.0 mm [10]. The bonded area (A) was calculated as:

$$\:A=\pi\:\left(\frac d2\right)2$$

resulting in a bonding area of 7.07 mm². SBS values were calculated using the following equation:

$$\:SBS=\:\frac{F\:max}{A}$$

where Fmax represents the maximum load (N) and A is the bonded area (mm²). Results were expressed in megapascals (MPa).

### Failure mode analysis

After SBS testing, fracture patterns at the interface between the 3D-printed substrate and the cement material were analyzed by digital microscopy (VHX series, Keyence, Osaka, Japan) at 50× magnification under standardized illumination conditions. Failure modes were classified according to the amount of repair material remaining on the substrate surface as follows: score 0, adhesive failure; score 1a, mixed failure type 1a (< 1/3 substrate coverage); score 1b, mixed failure type 1b (> 1/3 substrate coverage); and score 2, cohesive failure. To reduce subjectivity, all specimens were evaluated under the same optical conditions by a single calibrated examiner [10].

### Statistical analyses

Statistical analyses were performed using R software (R Foundation for Statistical Computing, Vienna, Austria; version 4.5.2). Data normality and homogeneity of variance were assessed prior to inferential analysis. For treated groups, surface roughness, flexural strength, elastic modulus, and SBS were analyzed using factorial ANOVA considering abrasive material (Al₂O₃ vs. SiO₂), air-abrasion time (5, 10, and 15 s), and aging condition (before vs. after), when applicable. The untreated control was analyzed as an additional reference group and compared with the treated groups using the same overall model followed by Tukey’s post hoc test. The significance level was set at α = 0.05.

## Results

### Surface roughness (Ra)

**Fig. 2:**
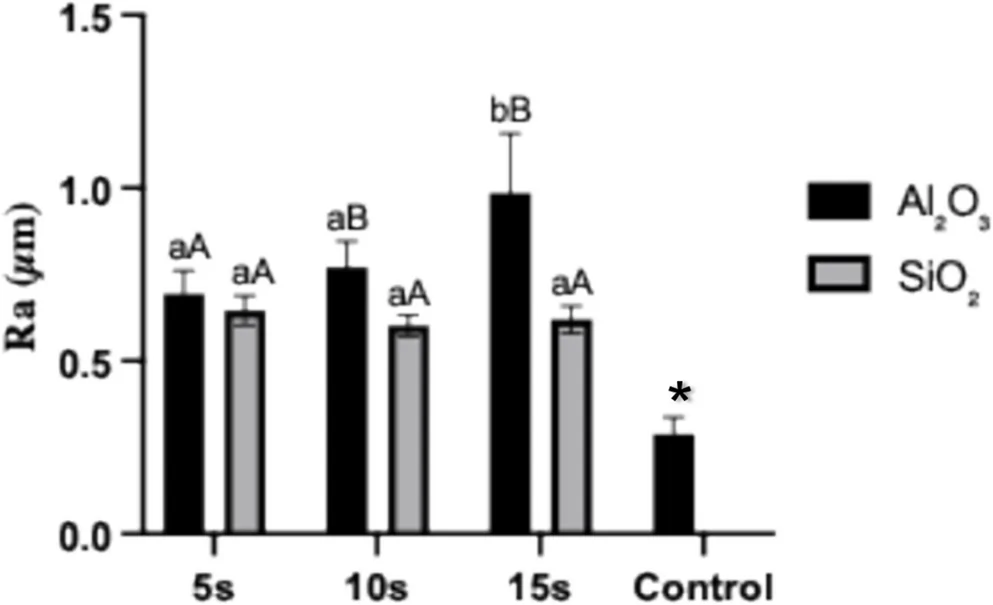
Surface roughness (Ra, µm) of specimens after different surface-conditioning protocols. Data are presented as mean ± standard deviation. Different lowercase letters indicate significant differences among the surface-conditioned groups (estimated marginal means with Sidak adjustment, α = 0.05). The untreated control was analyzed separately using Dunnett’s post hoc test and differed significantly from all surface-conditioned groups (*p* < 0.05)

Surface roughness (Ra) was significantly influenced by the surface-conditioning material (F = 7.97, *p* = 0.009, partial η² = 0.249), whereas neither application time (F = 1.39, *p* = 0.267) nor the interaction between material and application time (F = 2.59, *p* = 0.096) significantly affected the results (Supplementary Table S1). The untreated control group exhibited significantly lower Ra values than all surface-conditioned groups (Dunnett’s test, *p* < 0.05). Mean Ra values among the surface-conditioned groups ranged from 0.60 ± 0.05 μm (SiO₂ 10 s) to 0.77 ± 0.14 μm (Al₂O₃ 10 s). Multiple comparisons revealed significantly higher roughness for Al₂O₃ 10 s than SiO₂ 10 s (*p* < 0.05), whereas the remaining surface-conditioning protocols showed no significant differences (Fig. 2).

### Scanning electron microscopy (SEM)

**Fig. 3:**
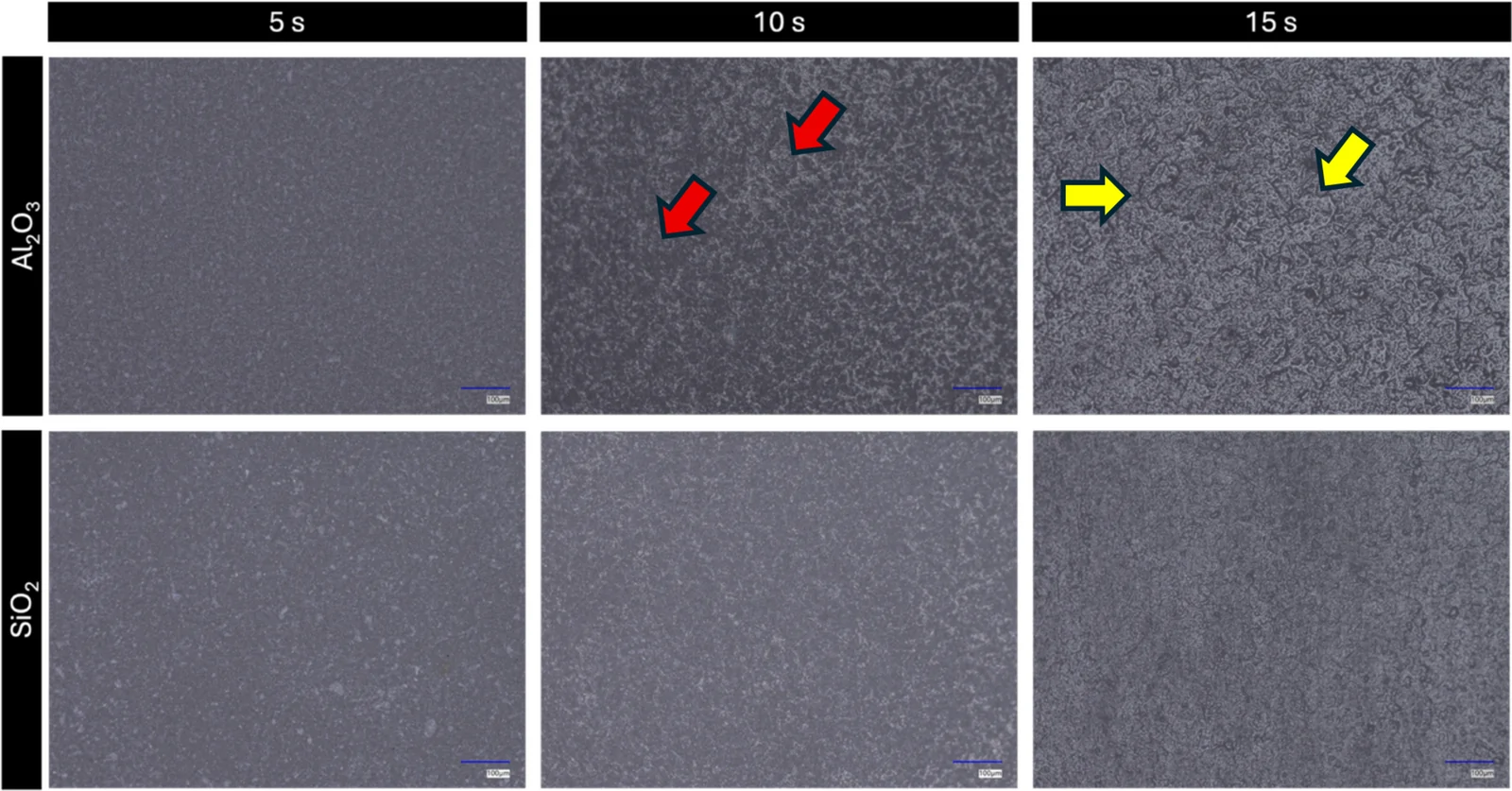
Representative SEM images of 3D-printed resin surfaces after air-abrasion using aluminum oxide (Al₂O₃) and tribochemical silica coating (SiO₂) at different application times (5, 10, and 15 s). Al₂O₃-treated surfaces exhibit a progressive increase in surface irregularity and defect density with increasing air-abrasion time, characterized by more pronounced topographical features and localized disruptions. In contrast, SiO₂-treated surfaces show a more homogeneous and uniform morphology across all time points, with less evident surface damage. Red arrows indicate localized surface depressions (valleys), whereas yellow arrows highlight microcrack formation. Scale bar: 100 μm

SEM observations revealed distinct surface morphologies depending on the abrasive material and application time (Fig. 3). Al₂O₃-treated specimens showed a clear time-dependent increase in surface irregularity. At 5 s, the surface appeared relatively smooth with limited disruption, whereas at 10 s, more pronounced topographical features became evident. At 15 s, the surface exhibited a highly irregular morphology, with increased heterogeneity and features consistent with cumulative particle impact and material removal. In contrast, SiO₂-treated specimens presented a more homogeneous surface across all time points. Although subtle surface modification was observed, the overall morphology remained comparatively uniform, with fewer pronounced irregularities and no evident progression of surface disruption with increasing application time.

### Flexural strength and elastic modulus

Three-way ANOVA revealed that thermocycling significantly affected flexural strength (F = 17.81, *p* < 0.001, partial η² = 0.186), whereas neither the surface-conditioning material (*p* = 0.355) nor the application time (*p* = 0.058) significantly influenced the results. None of the interaction terms reached statistical significance (all *p* > 0.05), indicating that the overall effect of thermocycling was independent of the surface-conditioning protocol and application time (Supplementary Table S2). The untreated control group exhibited significantly higher flexural strength than all surface-conditioned groups, regardless of the conditioning protocol or application time (Dunnett’s test, *p* < 0.05). No significant differences were observed among the surface-conditioned groups, regardless of the abrasive material (Al₂O₃ or SiO₂) or application time (5, 10, or 15 s) (*p* > 0.05). Pairwise comparisons demonstrated a significant reduction in flexural strength after thermocycling for the Al₂O₃ 5 s, SiO₂ 5 s, and SiO₂ 10 s groups, whereas no significant differences were observed for the remaining surface-conditioned groups (Table 1).

**Table 1:** Flexural strength (MPa) of specimens subjected to different surface conditioning and thermo-mechanical aging conditions

Group	Before (Mean ± SD)	After (Mean ± SD)
Al₂O₃ 5s	98.5 ± 5.5 A	85.2 ± 7.0 B
Al₂O₃ 10s	90.1 ± 11.8 A	82.6 ± 10.1 B
Al₂O₃ 15s	86.2 ± 14.1 A	83.4 ± 8.7 B
SiO₂ 5s	100.6 ± 12.4 A	85.6 ± 9.2 B
SiO₂ 10s	95.3 ± 8.6 A	82.4 ± 14.1 B
SiO₂ 15s	89.6 ± 7.8 A	85.1 ± 14.9 A
Control	117.1 ± 11.0*	106.2 ± 13.3*

* Data are presented as mean ± standard deviation. Different lowercase letters indicate significant differences among application times within the same surface-conditioning protocol, whereas different uppercase letters indicate significant differences between surface-conditioning protocols at the same application time (estimated marginal means with Sidak adjustment, *p* < 0.05). Thermocycling had no significant effect on elastic modulus (*p* > 0.05). The untreated control group was compared separately using Dunnett’s post hoc test; * indicates a significant difference from all surface-conditioned groups (*p* < 0.05)

Three-way ANOVA demonstrated that the type of surface-conditioning material significantly influenced the elastic modulus (F = 37.71, *p* < 0.001, partial η² = 0.326), representing the factor with the greatest effect on the outcome. Application time showed no significant main effect (*p* = 0.061), whereas thermocycling had no influence on elastic modulus (*p* = 0.777). A significant interaction between surface-conditioning material and application time was observed (F = 7.03, *p* = 0.002, partial η² = 0.153), indicating that the effect of application time depended on the surface-conditioning material. No significant interactions involving thermocycling were detected (Supplementary Table S3). The untreated control group exhibited significantly higher elastic modulus than all surface-conditioned groups, regardless of the conditioning protocol or application time (Dunnett’s test, *p* < 0.0001). Within the Al₂O₃-treated specimens, application time did not significantly affect the elastic modulus (all *p* > 0.05). In contrast, within the SiO₂-treated specimens, the 10 s and 15 s application times resulted in significantly higher elastic modulus values than the 5 s application (*p* = 0.049 and *p* = 0.0002, respectively), whereas no significant difference was observed between the 10 s and 15 s application times (*p* = 0.150). Comparison between surface-conditioning materials demonstrated no significant difference after 5 s of application (*p* = 0.524), whereas SiO₂ resulted in significantly higher elastic modulus than Al₂O₃ after 10 s and 15 s (both *p* < 0.0001). Thermocycling did not significantly affect the elastic modulus within any experimental group (all *p* > 0.05) (Table 2).

**Table 2:** Elastic modulus (GPa) of specimens subjected to different surface conditioning and thermo-mechanical aging conditions

Group	Before (Mean ± SD)	After (Mean ± SD)
Al₂O₃ 5 s	2.26 ± 0.24 aA	2.23 ± 0.14 aA
Al₂O₃ 10 s	2.07 ± 0.23 aA	1.99 ± 0.35 aA
Al₂O₃ 15 s	2.01 ± 0.22 aA	2.14 ± 0.24 aA
SiO₂ 5 s	2.40 ± 0.80 aA	2.33 ± 0.67 aA
SiO₂ 10 s	2.91 ± 1.04 bB	2.74 ± 0.51 bB
SiO₂ 15 s	3.16 ± 0.51 bB	3.21 ± 0.37 bB
Control	4.15 ± 0.14*	4.32 ± 0.31*

Data are presented as mean ± standard deviation. Different lowercase letters indicate significant differences among application times within the same surface-conditioning protocol, whereas different uppercase letters indicate significant differences between surface-conditioning protocols at the same application time (estimated marginal means with Sidak adjustment, *p* < 0.05). Thermocycling had no significant effect on elastic modulus (*p* > 0.05). The untreated control group was compared separately using Dunnett’s post hoc test; * indicates a significant difference from all surface-conditioned groups (*p* < 0.05)

### Weibull reliability analysis

The Weibull analysis provided additional information regarding the structural reliability of the tested materials beyond the comparison of mean flexural strength (Table 3). Before aging, the Al₂O₃ 5 s group exhibited the highest Weibull modulus (m = 19.64), indicating the most homogeneous flaw distribution among the surface-treated groups. Increasing the alumina air-abrasion time to 10 s and 15 s was associated with lower Weibull moduli (m = 7.97 and 8.39, respectively), suggesting greater variability in structural performance. In contrast, the tribochemical silica coating groups exhibited relatively stable Weibull moduli before aging (m = 12.70–16.50), indicating a more consistent structural response across the different application times.

**Table 3:** Reliability analysis of flexural strength according to a two-parameter Weibull distribution before and after thermocycling

Group	Before Aging			After Aging		
	Flexural strength (MPa)	m (95% CI)	σ₀ (95% CI)	Flexural strength (MPa)	m (95% CI)	σ₀ (95% CI)
Al₂O₃ 5 s	98.50 ± 5.53	19.64 (11.89–48.30)	101.00 (96.99-104.36)	85.24 ± 7.04	14.32 (8.41–32.22)	88.31 (82.67–92.31)
Al₂O₃ 10 s	90.05 ± 11.82	7.97 (4.82–31.64)	95.15 (86.00-103.85)	82.63 ± 10.14	9.13 (5.37–24.49)	87.00 (77.70–93.80)
Al₂O₃ 15 s	86.19 ± 14.13	8.39 (4.95–26.63)	91.49 (82.31–98.67)	83.39 ± 8.71	10.44 (6.01–29.25)	87.21 (79.71–93.41)
SiO₂ 5 s	100.61 ± 12.36	12.70 (7.27–46.92)	105.34 (97.44-109.44)	85.64 ± 9.16	9.61 (5.41–31.20)	89.71 (81.61–96.79)
SiO₂ 10 s	95.34 ± 8.62	13.39 (7.55–27.57)	99.08 (92.74-103.58)	82.44 ± 14.07	10.75 (3.70-71.56)	87.03 (78.22–91.63)
SiO₂ 15 s	90.26 ± 7.46	16.50 (9.56–37.45)	93.34 (88.40-96.66)	84.33 ± 14.79	8.56 (4.79–25.43)	89.58 (79.31–95.98)
Control	117.08 ± 10.97	15.53 (9.46–43.91)	121.51 (115.33-125.78)	106.20 ± 13.29	9.81 (5.78–22.38)	111.76 (101.68-119.59)

Reliability analysis of flexural strength of 3D-printed resin specimens subjected to different surface conditioning protocols before and after thermocycling according to a two-parameter Weibull distribution. Mean flexural strength (MPa), Weibull modulus (m), characteristic strength (σ₀), and their respective 95% confidence intervals (CI) obtained by bootstrap analysis are presented. The Weibull modulus (m) represents the structural reliability of the material, with higher values indicating a more homogeneous flaw population and lower variability in failure behavior. The characteristic strength (σ₀) corresponds to the stress level at which 63.2% of specimens are expected to fail according to the Weibull distribution

**Fig. 4:**
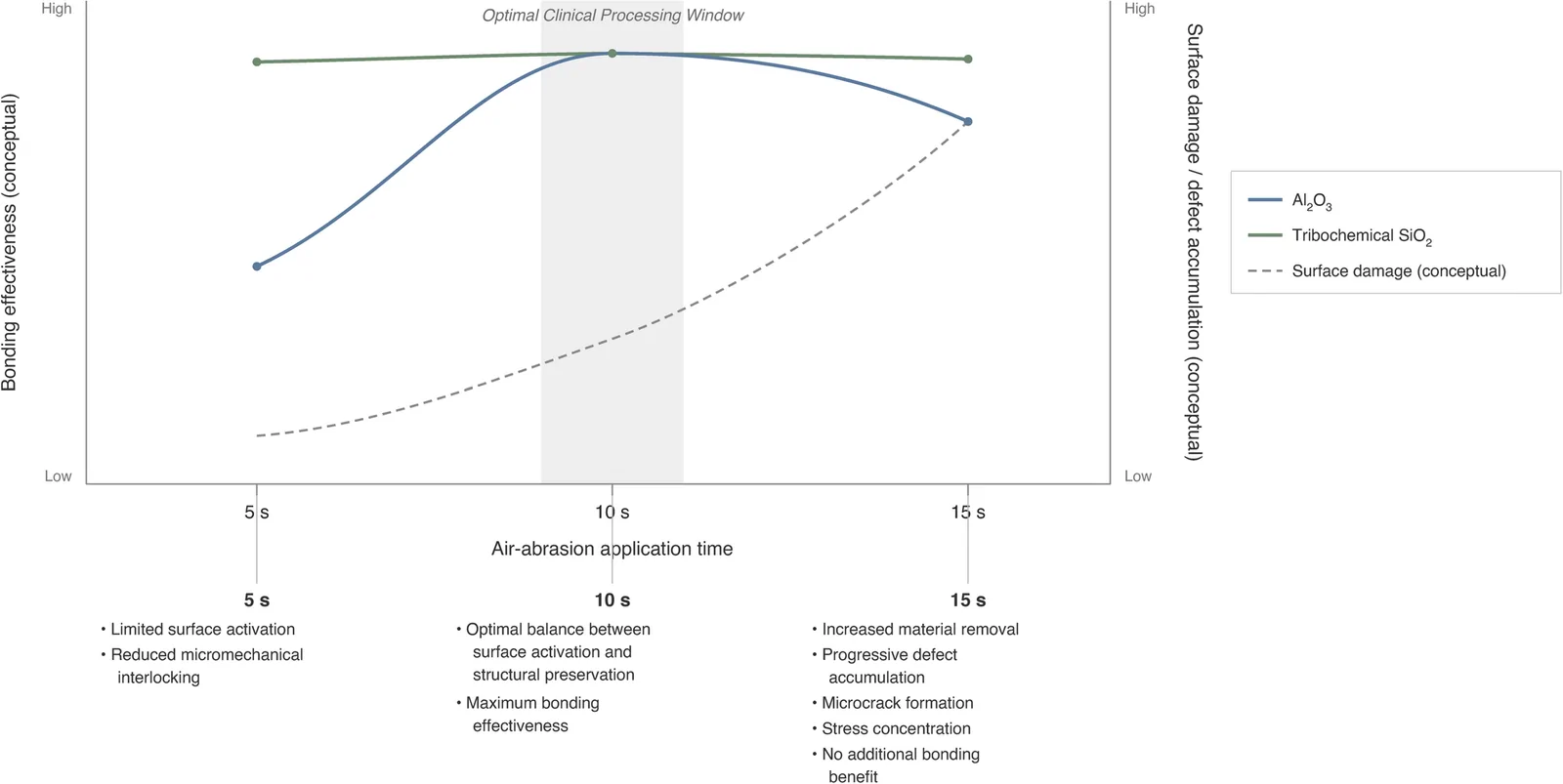
Conceptual representation of the clinical processing window for airborne-particle abrasion of 3D-printed resin-based materials. The diagram summarizes the balance between interfacial activation and structural damage as a function of air-abrasion application time. Aluminum oxide (Al₂O₃) is represented as a technique-sensitive surface treatment, in which bonding effectiveness increases up to an optimal application time but may decline with prolonged exposure because of progressive defect accumulation. In contrast, tribochemical silica coating (SiO₂) provides a more consistent bonding response over the evaluated application times. The dashed curve illustrates the conceptual increase in surface damage associated with longer air-abrasion exposure. The shaded region represents the proposed clinical processing window, in which adequate surface activation is achieved while minimizing structural damage

Following thermocycling, Weibull moduli decreased in most groups, indicating a reduction in structural reliability after aging. Characteristic strength (σ₀) followed the same general trend observed for mean flexural strength, with the control group presenting the highest values before (121.51 MPa) and after aging (111.76 MPa). Overall, the Weibull analysis suggests that prolonged alumina air abrasion may increase the variability of flaw distribution, whereas tribochemical silica coating tended to maintain a more homogeneous structural behavior across the evaluated application times. Based on the Weibull reliability analysis, a conceptual model illustrating the proposed clinical processing window for airborne-particle abrasion is presented in Fig. 4. The model summarizes the balance between surface activation and structural damage according to air-abrasion application time.

### Shear bond strength (SBS)

Three-way ANOVA revealed that both surface-conditioning material (F = 7.97, *p* = 0.006, partial η² = 0.069) and application time (F = 3.36, *p* = 0.039, partial η² = 0.059) significantly influenced shear bond strength, whereas thermocycling had no significant effect (F < 0.01, *p* = 0.959). None of the interaction terms were statistically significant (all *p* > 0.05), indicating that the effects of surface-conditioning material and application time were independent of thermocycling (Supplementary Table S4). The untreated control group exhibited significantly lower SBS values than all surface-conditioned groups, regardless of the conditioning protocol or application time (Dunnett’s test, *p* < 0.05). Within the Al₂O₃-treated groups, specimens treated for 5 s showed significantly lower SBS values than those treated for 10 s, whereas no significant difference was observed between the 10 s and 15 s application times. In contrast, SBS values of the SiO₂-treated groups were consistently high and did not differ significantly among the three application times (Table 4). Thermocycling did not significantly affect SBS within any experimental group (all *p* > 0.05).

**Table 4:** Shear bond strength (SBS) values (MPa) for the different experimental groups according to surface conditioning (Al₂O₃ or SiO₂), air-abrasion time (5 s, 10 s, and 15 s), and aging condition (before and after thermocycling)

Grupo	Before (Mean ± SD)	After (Mean ± SD)
Al₂O₃ 5s	10.52 ± 5.94 bcA	11.80 ± 4.03 bcA
Al₂O₃ 10s	17.86 ± 7.75 baA	17.28 ± 11.14 baA
Al₂O₃ 15s	19.23 ± 5.73 aA	10.98 ± 3.07 bcA
SiO₂ 5s	21.71 ± 12.97 aA	16.17 ± 8.35 aA
SiO₂ 10s	22.40 ± 10.58 aA	22.26 ± 11.59 aA
SiO₂ 15s	20.87 ± 10.68 aA	26.96 ± 12.36 aA
Control	5.72 ± 1.65 *	6.20 ± 2.64*

* Data are presented as mean ± standard deviation. Different lowercase letters indicate significant differences among application times within the same surface-conditioning protocol, whereas different uppercase letters indicate significant differences between surface-conditioning protocols at the same application time (estimated marginal means with Sidak adjustment, *p* < 0.05). Thermocycling had no significant effect on elastic modulus (*p* > 0.05). The untreated control group was compared separately using Dunnett’s post hoc test; * indicates a significant difference from all surface-conditioned groups (*p* < 0.05)

### Failure mode analysis

**Fig. 5:**
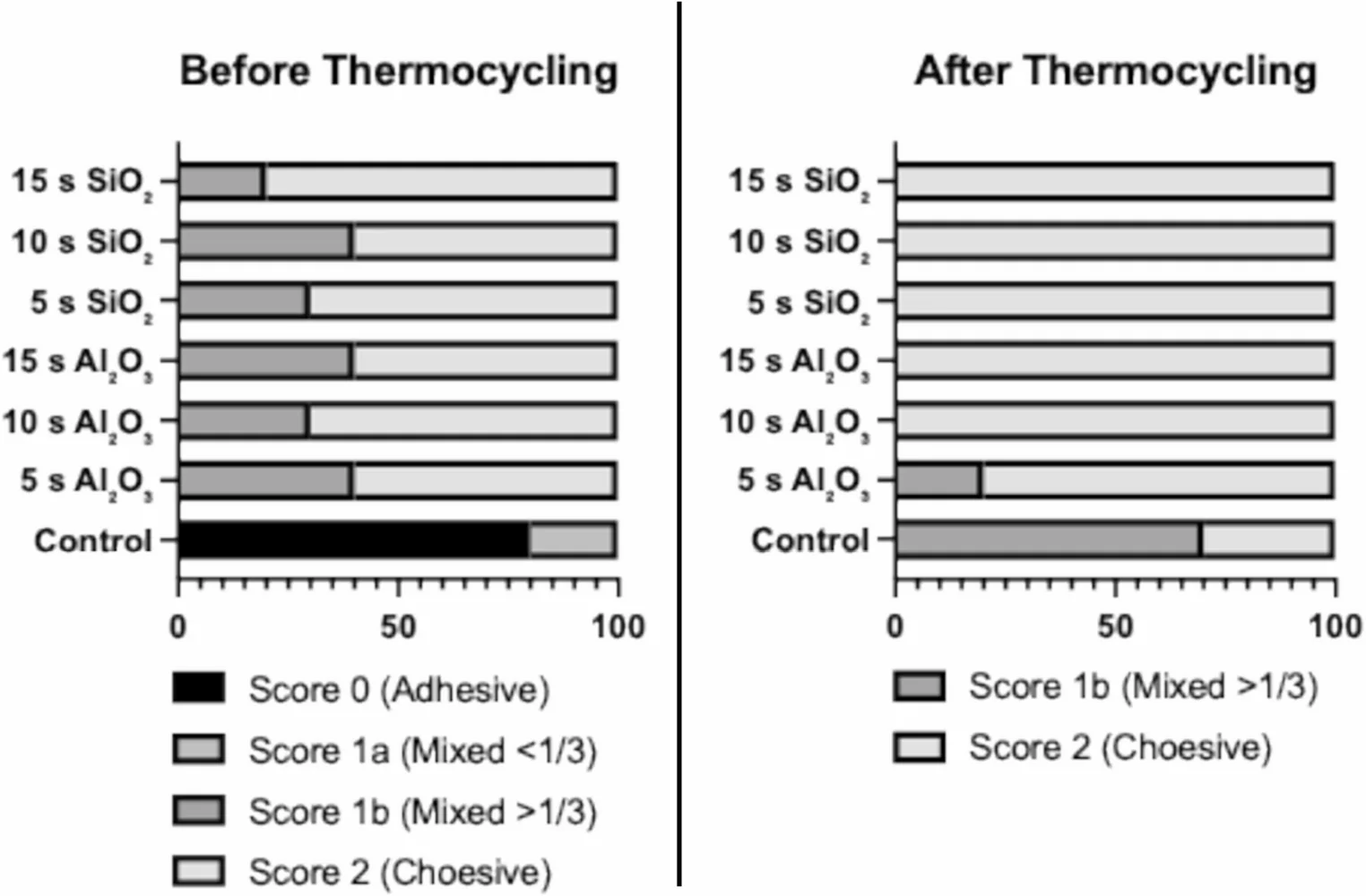
Distribution of failure modes (%) for each experimental group before and after thermo-mechanical aging. Failure patterns were classified as adhesive, mixed (types 1a and 1b), and cohesive

Failure mode analysis revealed a clear influence of surface conditioning and aging condition on fracture behavior (Fig. 5). Before thermocycling aging, the control group exhibited predominantly adhesive failures, whereas all surface-treated groups showed a transition toward mixed and cohesive failure patterns. Aluminum oxide and tribochemical silica treatments resulted in a higher proportion of cohesive failures, particularly with increasing air-abrasion time. After thermocycling aging, a marked shift in failure pattern was observed. All surface-treated groups exhibited predominantly cohesive failures, reaching up to 100% in several conditions. In contrast, the control group still presented a considerable proportion of mixed failures.

## Discussion

The present study investigated how different air-abrasion strategies affect the surface characteristics, mechanical behavior, and bonding performance of 3D-printed resin-based materials. The results clearly showed that surface conditioning cannot be interpreted as a purely topographical modification. Instead, it represents a balance between surface activation and defect generation, with direct implications for both interfacial performance and structural integrity [2, 5]. Based on these findings, the null hypotheses were partially rejected. Surface conditioning and air-abrasion time significantly affected multiple outcomes, including surface roughness, flexural strength, and bond strength, whereas the effect of aging was limited and property-dependent. Moreover, interaction effects were observed for specific parameters, indicating that the influence of air-abrasion time depends on the abrasive material used.

Surface conditioning significantly affected the evaluated properties, whereas the influence of application time was outcome-dependent. Aging showed a limited and property-dependent effect, being significant only for flexural strength. As expected, Al₂O₃-treated specimens exhibited a time-dependent increase in roughness, reflecting the cumulative effect of particle impact and material removal. This behavior is consistent with the intrinsic morphology of alumina particles, which are typically angular and sharp-edged, promoting localized cutting and plastic deformation [17]. In contrast, tribochemical silica coating resulted in a more stable surface profile across all time points. This difference likely arises from the distinct mechanism of interaction, where silica-coated particles promote a more controlled modification, with limited material removal and partial deposition of a silica-rich layer. While increased roughness is often associated with improved adhesion, excessively irregular surfaces may hinder complete wetting by resin cements. Deep valleys may not be fully infiltrated, potentially leading to void formation and stress discontinuities at the interface. Therefore, roughness should be interpreted not only in terms of magnitude, but also in terms of morphology and accessibility [18].

SEM observations support this interpretation. Alumina-treated surfaces exhibited pronounced irregularities, and features consistent with cumulative particle impact, whereas silica-treated surfaces showed a more homogeneous morphology with fewer apparent defects. From a mechanical perspective, this difference is highly relevant. Irregular geometries such as sharp valleys and microcracks act as stress concentrators, locally amplifying applied stresses. According to fracture mechanics principles, these regions exhibit higher stress intensity, facilitating crack initiation under loading [10, 19]. More uniform surfaces, on the other hand, tend to distribute stresses more evenly, reducing localized amplification. This becomes particularly evident when considering flexural strength. All surface-treated groups showed reduced strength compared to the control, regardless of abrasive type or exposure time. This indicates that even short air-abrasion times are sufficient to introduce defects that dominate mechanical behavior. From a fracture mechanics perspective, failure is controlled by the most critical flaw, and surface defects act as crack initiation sites under tensile stress [9, 10].

At the same time, it is important to contextualize these findings clinically. In practice, abraded surfaces are subsequently covered by a resin cement layer, which can infiltrate surface irregularities and partially redistribute stresses. This interfacial layer may reduce the effect of superficial defects by improving load transfer and filling accessible voids. However, subsurface defects and microcracks introduced during air-abrasion are not eliminated by cementation [6, 20]. Although the manufacturer recommends 110-µm Al₂O₃ particles for Crowntec restorations, 50-µm particles were intentionally used to more closely approximate the dimensions of the particles employed by the tribochemical silica-coating system. This choice aimed to reduce particle size as a potential confounding factor and to provide a more controlled comparison of the effects of conditioning system and application time. Nevertheless, because the two commercially available abrasive systems do not have identical particle dimensions or compositions, the findings should be interpreted as a comparison between surface-conditioning protocols rather than as an isolated comparison of abrasive chemistry.

The elastic modulus followed a similar trend, with higher values observed in the control group. Although modulus is less sensitive to surface defects than strength, the introduction of heterogeneity and local damage can still affect the material’s resistance to deformation [14].

The bond strength results reinforce the idea that the system cannot be interpreted solely based on the type of abrasive. Although a significant effect of material was observed, no consistent superiority was found across all conditions. Instead, the most evident pattern was related to application time, particularly for Al₂O₃. Increasing the air-abrasion time from 5 s to 10 s improved bond strength, suggesting that a minimum level of surface activation is required. However, extending the treatment to 15 s did not provide additional benefit and tended to reduce values after aging. This behavior reflects a transition from beneficial surface modification to defect accumulation [4, 5, 21].

In contrast, silica-coated surfaces showed a more stable response across time intervals, with less sensitivity to changes in exposure time. This suggests that the tribochemical mechanism provides a more controlled interfacial condition, combining mechanical interaction with chemical compatibility [8, 22]. However, it is important to emphasize that this does not necessarily represent superior performance, but rather a more consistent response under varying conditions.

The intrinsic characteristics of the substrate also contribute to this behavior. Unlike conventional resin composites, zirconia, or glass-ceramics, which present higher filler content and superior mechanical properties, 3D-printed resins are typically more polymer-rich and mechanically less resistant. As a result, they are more susceptible to defect-driven degradation [9, 20]. In this context, aggressive surface conditioning or excessive air-abrasion times may not immediately compromise bond strength but can negatively affect long-term performance by weakening the material and increasing its sensitivity to fatigue and aging.

Failure mode analysis corroborates the bond strength findings and provides additional insight into the fracture behavior of the interface. Higher bond strength values were consistently associated with mixed and predominantly cohesive failures, indicating effective stress transfer across the bonded interface [6, 23]. In contrast, the control group, which exhibited lower bond strength values, showed a predominance of adhesive failures prior to aging, reflecting a weak and unstable interface. After thermocycling aging, a clear shift toward cohesive failures was observed in all surface-treated groups, in some cases reaching 100%, suggesting that the interfacial strength exceeded the cohesive strength of the substrate or repair material. This transition indicates that failure was no longer governed by interfacial debonding, but rather by the intrinsic mechanical properties of the material. Importantly, the occurrence of cohesive failures within the substrate highlights that, once an adequate surface conditioning is achieved, the integrity of the bulk material becomes the limiting factor for overall performance.

In this study, the aging protocol was limited to 5,000 thermocycles, which may not fully capture long-term degradation mechanisms, particularly those related to subcritical crack growth [20]. In addition, only one type of 3D-printed resin was evaluated, and variations in composition, printing parameters, and post-curing conditions may influence the response to surface conditioning.

Future studies should focus on the long-term implications of surface-induced defects, particularly under more aggressive aging conditions, including extended thermocycling and mechanical fatigue. Approaches such as fractographic analysis and advanced imaging techniques may provide further insight into defect distribution and crack propagation mechanisms [19]. From a clinical standpoint, optimizing surface conditioning protocols requires balancing interfacial performance and structural preservation, especially in materials with inherently lower mechanical resistance.

## Conclusions

From this study, the following conclusions can be drawn:

- Tribochemical silica coating produced significantly higher shear bond strength than with aluminum oxide air-abrasion and maintained the mechanical properties of the printed resin more consistently than aluminum oxide air-abrasion.
- Higher surface roughness did not necessarily result in higher bond strength.
- Thermocycling reduced flexural strength but did not significantly affect shear bond strength or elastic modulus.
- Under the experimental conditions evaluated, tribochemical silica coating applied for 10 s achieved comparable performance to 15 s, indicating that longer application times are unnecessary.

## Supplementary Information

Below is the link to the electronic supplementary material.

Supplementary Material 1. Supplementary Table S1. Two-way ANOVA and effect size analysis for roughness. Supplementary Table S2. Three-way ANOVA and effect size analysis for flexural strength. Supplementary Table S3. Three-way ANOVA for shear bond strength. Supplementary Table S4. Three-way ANOVA for shear bond strength.

## Data Availability

No datasets were generated or analysed during the current study.
