# Supplementary material for: Effect of air-borne particle abrasion and tribochemical silica coating on surface characteristics, mechanical behavior, and bonding performance of 3D-printed resin-based materials
Source: Clin Oral Investig. 2026 Sep 12;30(10):441. doi: 10.1007/s00784-026-07123-z (PMC13570911; doi:10.1007/s00784-026-07123-z)
Supplement: Supplementary file 1 [file 784_2026_7123_MOESM1_ESM.pdf]

Supplementary

Supplementary Table S1. Two-way ANOVA and effect size analysis for roughness.

Source	df	F	p	Partial η² (95% CI)
Material	1	7.97	0.009	0.249 (0.031–0.460)
Application time	2	1.39	0.267	0.104 (0.000–0.311)
Material × Application time	2	2.59	0.096	0.177 (0.000–0.392)

Supplementary Table S2. Three-way ANOVA and effect size analysis for flexural strength.

Source	df	F	p	Partial η² (95% CI)
Material	1	0.87	0.355	0.011 (0.000–1.000)
Application time	2	2.95	0.058	0.070 (0.000–1.000)
Thermocycling	1	17.81	<0.001	0.186 (0.073–1.000)
Material × Time	2	0.04	0.965	0.001 (0.000–1.000)
Material × Thermocycling	1	0.58	0.449	0.007 (0.000–1.000)
Time × Thermocycling	2	1.56	0.216	0.039 (0.000–1.000)
Material × Time × Thermocycling	2	0.06	0.943	0.002 (0.000–1.000)

Supplementary Table S3. Three-way ANOVA for shear bond strength.

Source	df	F	p	Partial η² (95% CI)
Material	1	37.71	<0.001	0.326 (0.191–1.000)
Application time	2	2.89	0.061	0.069 (0.000–1.000)
Thermocycling	1	0.08	0.777	0.001 (0.000–1.000)
Material × Time	2	7.03	0.002	0.153 (0.041–1.000)
Material × Thermocycling	1	0.10	0.753	0.001 (0.000–1.000)
Time × Thermocycling	2	0.32	0.728	0.008 (0.000–1.000)
Material × Time × Thermocycling	2	0.01	0.995	<0.001

Supplementary Table S4. Three-way ANOVA for shear bond strength.

Source	df	F	p	Partial η² (95% CI)
Surface-conditioning material	1	7.97	0.006	0.069 (0.006–0.177)
Application time	2	3.36	0.039	0.059 (0.000–0.154)
Thermocycling	1	0.00	0.959	<0.001 (0.000–0.009)
Material × Time	2	0.51	0.601	0.009 (0.000–0.061)
Material × Thermocycling	1	0.00	0.981	<0.001 (0.000–0.000)
Time × Thermocycling	2	0.29	0.746	0.005 (0.000–0.047)
Material × Time × Thermocycling	2	0.64	0.528	0.012 (0.000–0.068)
